# Novel De Novo DLL4 Missense and Highly Accurate Protein Structure Prediction in Adams–Oliver Type 6 Syndrome

**DOI:** 10.1002/ccr3.70933

**Published:** 2025-10-01

**Authors:** Rodrigo Cabrera, Marlon Yesid Barrera Montañez, Sebastian Ramiro Gil‐Quiñones, Adriana Motta Beltrán, Natalia Santiago‐Tovar, Nora Contreras‐Bravo, Dora Janeth Fonseca‐Mendoza, Carlos Martin Restrepo, Adrien Morel

**Affiliations:** ^1^ School of Medicine and Health Sciences, Center for Research in Genetics and Genomics (CIGGUR), Institute of Translational Medicine (IMT) Universidad del Rosario Bogotá Colombia; ^2^ Laboratorio de Biología Molecular y Pruebas diagnósticas de Alta Complejidad Fundación Cardioinfantil—Instituto de Cardiología Bogotá Colombia; ^3^ Departamento de Dermatología Universidad El Bosque Bogotá Colombia

**Keywords:** Adams–Oliver syndrome type 6, alphafold, artificial intelligence, DLL4, PremPS, WES

## Abstract

Adams–Oliver syndrome (AOS) is a rare disease classically described with scalp vertex aplasia cutis and terminal transverse limb defects. This syndrome is frequently misdiagnosed by taking each feature of the disease separately. A novel *de novo* missense variant in *DLL4* (c.998G>A, p.Cys333Tyr) was identified by Whole Exome Sequencing (WES), and structural analysis using AlphaFold and PremPS confirmed its pathogenicity by disrupting the NOTCH1 signaling pathway, highlighting the power of AI‐driven tools in variant interpretation.

## Graphical abstract

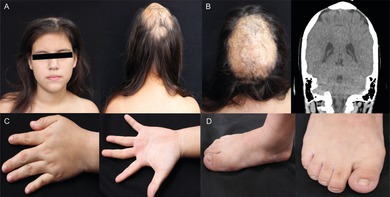

## 1 Introduction

Adams–Oliver syndrome (AOS) is a rare inherited disorder classically described with scalp vertex aplasia cutis and terminal transverse limb defects [1, 2]. Vascular abnormalities such as congenital telangiectatic cutis marmorata, pulmonary and portal hypertension, and retinal hypervascularization are been seen [3]. Additionally, congenital heart defects are present in 20% of cases, primarily including ventricular septal defects, valve abnormalities, great vessel anomalies, and Tetralogy of Fallot.

AOS exhibits genetic and mutational heterogeneity with both autosomal dominant and recessive modes of inheritance [2]. Autosomal dominant inheritance has been linked to mutations in *ARHGAP31* (AOS1), *RPBJ* (AOS3), *NOTCH*1 (AOS5), and *DLL4* (AOS6) genes, while autosomal recessive inheritance is associated with mutations in *DOCK6* (AOS2) and *EOGT* (AOS4) genes (OMIM #100300, #614219, #614814, #615297, #616028, and #616589, respectively). Molecular analysis is crucial for establishing a precise genetic diagnosis, elucidating genotype–phenotype correlations, risk assessment, and genetic counseling.

## 2 Methods (Differential Diagnosis, Investigations and Treatment)

### 2.1 Case Presentation

**FIGURE 1:**
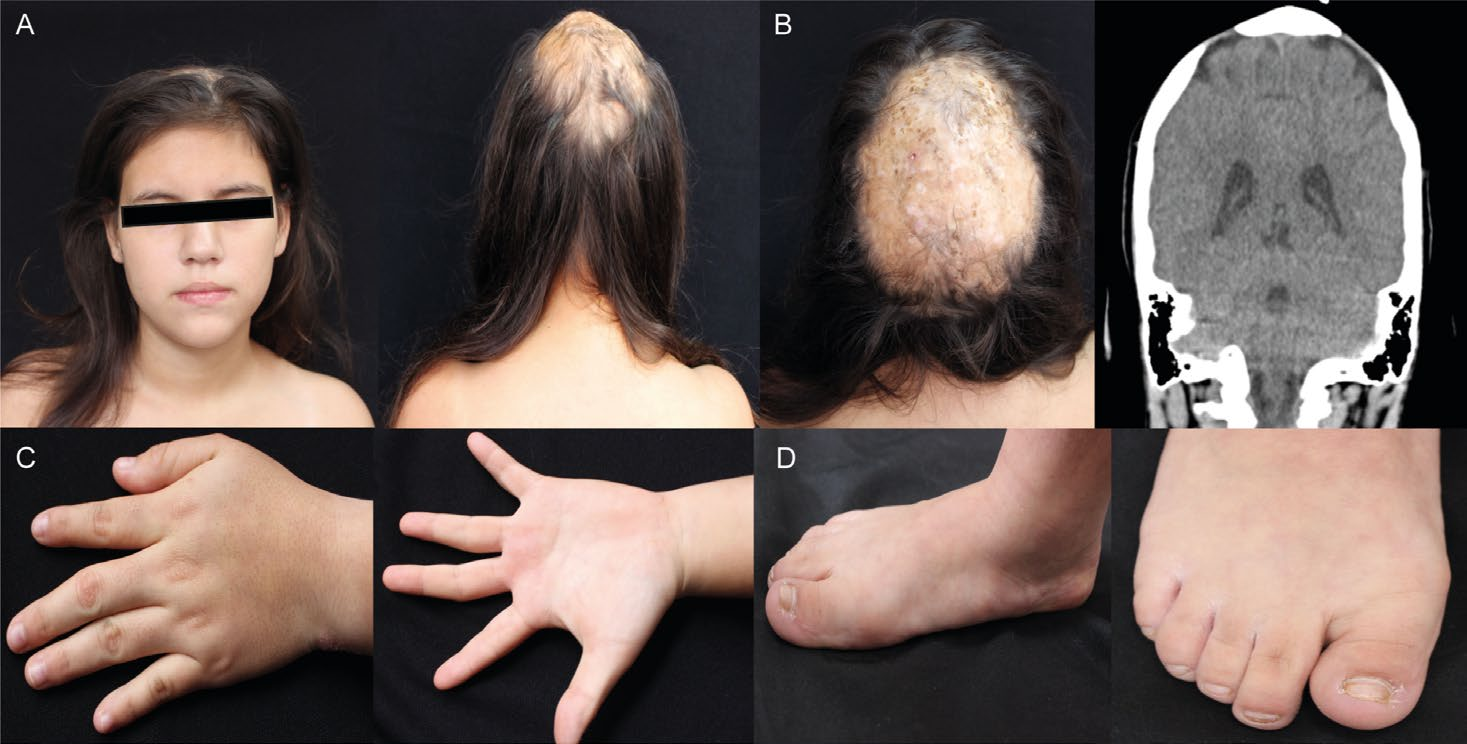
Adams–Oliver type 6 Patient: (A) Front and back: Fitzpatrick phototype III patient with dolichocephaly and craniosynostosis. (B) Skin and skull: Scalp with atrophic and alopecic areas, and CT scan showing bitemporal bone anomalies. (C) Left hand: Mild syndactyly (3rd and 4th fingers) and symbrachydactyly. (D) Right toe: Hypoplastic nails.

A 13‐year‐old girl was admitted to the emergency unit with high‐intensity holocranial headache and upper limb paresthesias. Physical examination revealed dolichocephaly, craniosynostosis, epilepsy, alopecia, and atrophic scalp skin (Figure 1A,B). At birth, her scalp was fragile and ulcerated, and she had a wide, smooth fontanel. Her parents were healthy and nonconsanguineous, and her two older siblings and other relatives showed no signs of the disease. Clinical examination showed biparietal scalp atrophic skin, wide alopecic areas on the vertex, surface irregularities in the underlying bone structure, mild bilateral lower extremity syndactyly, brachydactyly, and hypoplastic nails (Figure 1C,D).

### 2.2 Differential Diagnosis

Brain imaging was performed to rule out major structural brain malformations that could be associated with syndromic craniosynostosis or other genetic syndromes involving neurological abnormalities. Both cranial CT and MRI confirmed multiple calvarial bone defects but did not reveal additional brain malformations (Figure 1B). These findings helped differentiate AOS from other craniosynostosis syndromes with neurological involvement.

Assessment of cardiac abnormalities was necessary due to the known association of AOS with congenital heart defects. Echocardiographic evaluation ruled out structural cardiac anomalies, further supporting the distinction from syndromes that frequently involve cardiovascular abnormalities.

Based on phenotype alone, it was not possible to determine the specific AOS subtype, highlighting the necessity of genetic testing to establish a precise diagnosis.

## 3 Conclusion and Results (Outcome and Follow‐Up)

**FIGURE 2:**
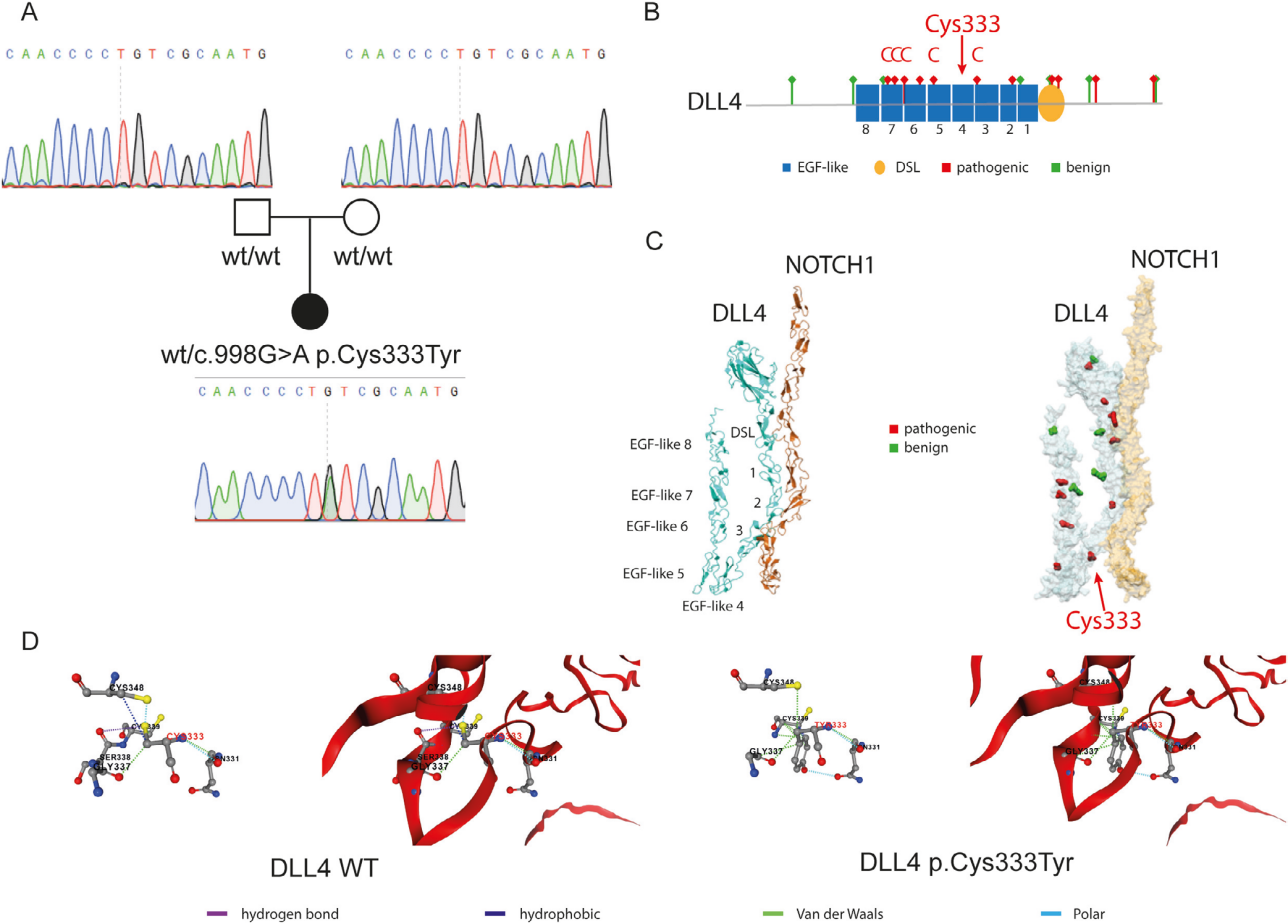
*DLL4* gene sequencing and AI predictions: (A) Genealogic tree and chromatograms of Sanger sequencing (B) Secondary structure of DLL4 protein with benign (green) and pathogenic (red) variants (C) Tertiary structure of heterodimer DLL4/NOTCH1 proteins with benign (green) and pathogenic (red) variants thanks to Alphafold predictions (D) Atomic interactions of the interested amino acid (Cysteine 333 left (WT) or Tyrosine 333 right (patient mutation)) with PremPS predictions.

Whole Exome Sequencing (WES) identified 326,124 variants in the patient's exome. After filtering for variants with a minor allele frequency (MAF) ≤ 0.01 and using a virtual gene panel composed of genes associated with AOS (*ARHGAP31, DLL4, DOCK6, EOGT, NOTCH1*, and *RBPJ*), five candidate variants were identified. Among them, a novel de novo heterozygous missense variant in *DLL4* (c.998G>A, p.Cys333Tyr) was determined to be pathogenic. This variant was absent in both parents, confirming its *de novo* origin (Figure 2A, Figures S1 and S2).

To further assess the impact of this mutation, we performed structural modeling using AlphaFold‐multimer (v.2.3.0) to predict the interaction of DLL4 with NOTCH1. The structural prediction suggested that the p.Cys333Tyr variant disrupted key disulfide bond formations critical for the function of DLL4. The predicted Cys333Tyr variant is the first reported variant affecting a cysteine in the EGF‐like 4 domain of DLL4 (Figure 2B,C). Additionally, PremPS analysis indicated a destabilizing effect on protein interactions, supporting its pathogenic nature: the cysteine had 2 polar, two van der Waals, and 1 hydrophobic and 1 hydrogen bond, while the tyrosine had 7 van der Waals and 2 polar bonds (Figure 2D).

Pathogenic variants previously reported in ClinVar database (https://www.ncbi.nlm.nih.gov/clinvar/) have been found affecting multiple residues within the MNNL and DSL domains, as well as an exceptional concentration of variants affecting cysteine residues in EGF‐like domains 3–7 of DLL4. The identification of a pathogenic cysteine‐substituting variant in the EGF‐like 4 domain of DLL4 aligns with this pattern, reinforcing its probable role in disease pathogenesis (Figure 2C).

Segregation analysis was performed using Sanger sequencing with specific primers (huDLL4_For: GACTTTGAGTTGAGGTGTCTTTGA and huDLL4_Rev: CACTAACTGCCTAGGTTAGGGATG), confirming the absence of the variant in both parents (Figure 2A). This further reinforced the classification of this variant as a *de novo* mutation.

The combination of WES, structural protein modeling using AlphaFold, and interaction analysis via PremPS provides strong evidence supporting the pathogenicity of this novel *DLL4* variant. These findings suggest a direct impact on NOTCH1 pathway signaling, reinforcing its role in the molecular pathology of AOS type 6.

## 4 Discussion

Clinical and dermatological features of AOS exhibit a wide range of anomalies, and a potential correlation between genotype and phenotype has been suggested [4]. Aplasia cutis congenita (ACC) (81%) and transverse terminal limb defects (TTLD) (61%) are strong indicators of the disease, particularly in cases with a positive family history [3, 4, 5]. Cutis marmorata telangiectatica congenita (CMTC) has been reported in up to 62% of cases [5, 6]. Despite the presence of six genetic subtypes, each caused by a specific gene: *ARHGAP31*, *DLL4*, *DOCK6*, *EOGT*, *NOTCH1*, and *RBPJ* (OMIM #100300, #614219, #614814, #615297, #616028, and #616589), clinical differentiation is often challenging, necessitating molecular analysis for accurate diagnosis; WES is a molecular tool that allows for the easy identification of mutations in the specific gene associated with the clinical subtype [7, 8]. In our patient, we found a novel heterozygous *de novo* missense on the *DLL4* gene related to Adams–Oliver type 6 syndrome (AOS6).

DLL4 plays a critical role in angiogenesis with Vascular Endothelial Growth Factor (VEGF) and the DLL4‐NOTCH1 signaling [9, 10]. The identified Cys333Tyr variant, along with the overrepresentation of cysteine‐affecting variants in DLL4, suggests a mechanism similar to that observed in Alagille syndrome, CADASIL, and Marfan syndrome, where pathogenicity is linked to cysteine substitutions within epidermal growth factor (EGF)‐like domains [11, 12, 13, 14]. However, the involvement of cysteine residues in DLL4‐related AOS6 has not been previously described. As previously observed in the homologous rat complex, this study predicts an interaction between the MNNL and DSL domains of DLL4 protein with 11 and 12 of Notch1 EGF receptor domains, allowing us to identify structural patterns in the localization of known pathogenic mutations [15].

Structural predictions using AlphaFold and PremPS highlight the potential impact of the Cys333Tyr variant on DLL4‐NOTCH1 interactions, strengthening its classification as a pathogenic mutation. These findings align with previous studies demonstrating that artificial intelligence‐based protein modeling can enhance our understanding of rare genetic disorders [16, 17].

By leveraging AI‐driven structural predictions, we can refine variant classification and improve genetic counseling strategies. The use of computational tools such as AlphaFold and PremPS offers a promising avenue for elucidating pathogenic mechanisms in genetic diseases, potentially reducing the need for extensive functional assays. Our findings provide further evidence of the importance of integrating molecular analysis and AI‐driven structural predictions in the diagnosis and characterization of rare syndromes like AOS6.

## Author Contributions

**Rodrigo Cabrera:** conceptualization, data curation, formal analysis, investigation, software, validation, writing – original draft, writing – review and editing. **Marlon Yesid Barrera Montañez:** conceptualization, formal analysis, investigation, supervision, validation, visualization, writing – original draft, writing – review and editing. **Sebastian Ramiro Gil‐Quiñones:** conceptualization, data curation, formal analysis, investigation, supervision, validation, visualization, writing – original draft, writing – review and editing. **Adriana Motta Beltrán:** conceptualization, data curation, formal analysis, investigation, supervision, validation, visualization, writing – original draft, writing – review and editing. **Natalia Santiago‐Tovar:** conceptualization, data curation, investigation, validation, writing – original draft, writing – review and editing. **Nora Contreras‐Bravo:** investigation, methodology, validation, visualization, writing – review and editing. **Dora Janeth Fonseca‐Mendoza:** investigation, methodology, validation, visualization, writing – review and editing. **Carlos Martin Restrepo:** conceptualization, data curation, formal analysis, investigation, methodology, supervision, validation, visualization, writing – original draft, writing – review and editing. **Adrien Morel:** conceptualization, data curation, formal analysis, investigation, methodology, software, supervision, validation, visualization, writing – original draft, writing – review and editing.

## Consent

Written informed consent was obtained from the patient to permit the publication of this report.

## Conflicts of Interest

The authors declare no conflicts of interest.

## Supporting information


**Figure S1:** DLL4/NOTCH pathway involved in Adams–Oliver syndrome. The pathogenic variant is located on an interaction site between DLL4 and NOTCH1 (predicted by AI), which leads to inhibition of the NOTCH pathway and the development of the Adams–Oliver syndrome.
**Figure S2:** Schematic overview of AlphaFold2 protein structure prediction.

## Data Availability

All data relevant to the study are included in the article. Additional data are available from the corresponding author upon request.
