# Supplementary material for: Novel De Novo DLL4 Missense and Highly Accurate Protein Structure Prediction in Adams–Oliver Type 6 Syndrome
Source: Clin Case Rep. 2025 Oct 1;13(10):e70933. doi: 10.1002/ccr3.70933 (PMC12485272; doi:10.1002/ccr3.70933)
Supplement: Supplementary file 1 — Figure S1: DLL4/NOTCH pathway involved in Adams–Oliver syndrome. The pathogenic variant is located on an interaction site between DLL4 and NOTCH1 (predicted by AI), which leads to inhibition of the NOTCH pathway and the development of the Adams–Oliver syndrome. Figure S2: Schematic overview of AlphaFold2 protein structure prediction. [file CCR3-13-e70933-s001.docx]

**Supplementary Material**

1. **Whole Exome Sequencing**

DNA was extracted using the DNA Miniprep kit (ZYMO Research, USA) according to the manufacturer's protocol. The library was constructed from 250 ng of DNA with the MGIEasy FS DNA Library Prep kit and DNA was fragmented by enzymes approximately 200-400bp. The fragmented DNA was repaired, amplified by PCR and captured by Exome Capture V5 Probe with streptavidin beads and specific primers were used to amplify and enrich the regions of interest. A circularization of the DNA was realized and then a denaturation of the library after ligation of Split-oligo and digestion and purification by specific beads. The circulated DNA was used to generate nanoballs (DNB), which consist in a cyclic amplification through Rolling Circle Amplification process. DNB were quantified and charged into a flow cell (FCL_PE100) to be sequenced by DNBseqG400. After sequencing, the quality of data was 90% with Q30.

The reads were mapped to the reference genome hg19 using BWA (Burrows-Wheeler Aligner) and sorted by SAMtools. Duplicated reads were eliminated by Picard software. Variants were identified with Haplotyper (Sentieron) software v4.0.5.1. Depth and breadth of coverage were analyzed by BAMBA software to achieve a depth of 50x. VCF file was analyzed by VarSeq software v2.3.0 (GoldenHelix). Variants were filtered by MAF ≤0.01 and a virtual gene panel composed of the genes: *ARHGAP31*, *DLL4*, *DOCK6*, *EOGT*, *NOTCH1* and *RBPJ*. Variant confirmation and segregation analysis were performed through Sanger sequencing using specific primers: huDLL4_For: gactttgagttgaggtgtctttga and huDLL4_Rev: cactaactgcctaggttagggatg. The chromatogram was analyzed by Snapgene viewer.

1. **Structural analysis of DLL4-NOTCH1 complex**

The structure of the DLL4-Notch1 complex was predicted using AlphaFold-multimer (v.2.3.0), using default settings, with the exception of the --reduced_dbs flag to reduce storage space requirements. Initially, the entire cytoplasmic domains of both proteins (excluding signal peptides) were used for the prediction. This prediction mapped the interactions to EGF domains 11 and 12 in Notch1, and this information was used to predict an additional structure using the entire cytoplasmic domain of DLL4, but only EGF domain 10-14 in notch1. This simplified model was used to show the different features of the predicted structure for DLL4-Notch1 (Figure S2).

1. **Structural mapping of known *DLL4* mutations**

Known variants in *DLL4*, classified as pathogenic, likely pathogenic, likely benign or benign were extracted from the ClinVar database in May 2023. These variants were mapped to a domain structure model of the DLL4 protein using the MyDomains tool at <https://prosite.expasy.org/mydomains> (Figure S2).

1. **Interaction amino acid evaluation by PremPS**

DLL4 structure PDB (AF-Q9NR61-F1-model_v4) was used from Alphafold to predict the impact of the mutation on interaction amino acid thanks to PremPS (<https://lilab.jysw.suda.edu.cn/research/PremPS/>) (Figure S2).


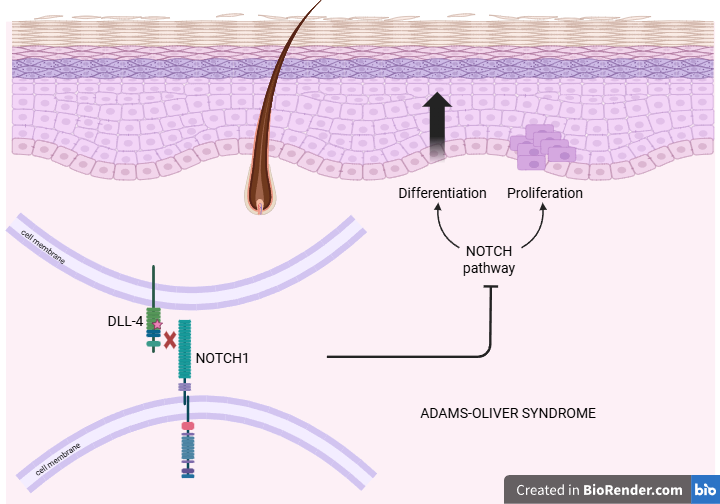


**Figure S1. DLL4/NOTCH pathway involved in Adams-Oliver syndrome.** The pathogenic variant in our study is located on an interaction site between DLL4 and NOTCH1 (predicted by AI), which leads to inhibition of NOTCH pathway and the development of the Adams-Oliver syndrome.


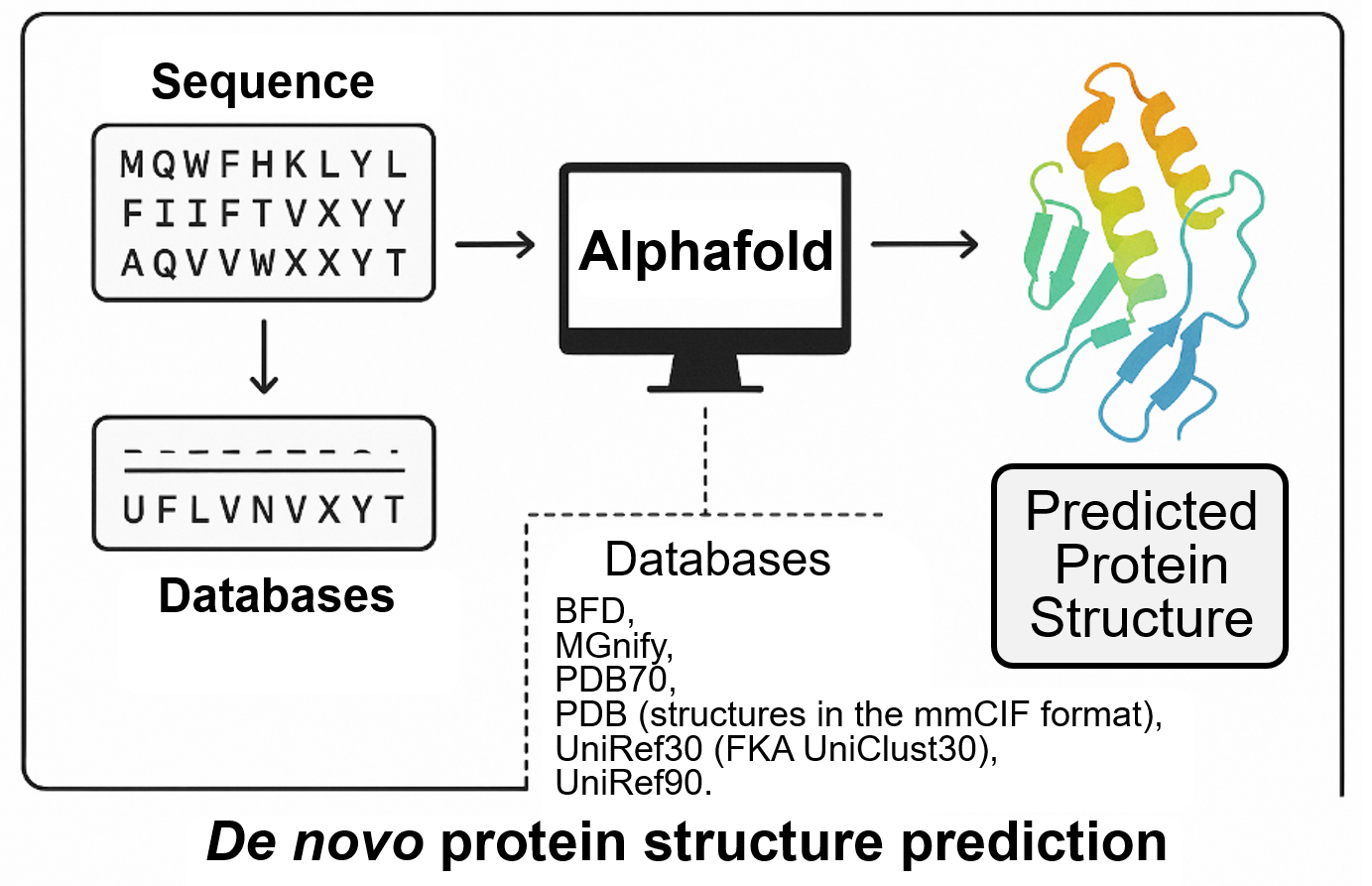


**Figure S2. Schematic overview of AlphaFold2 protein structure prediction.**

AlphaFold2 predicts the 3D structure of a protein from its amino acid sequence. The process begins with the input of a target protein sequence, followed by a sequence homology search across multiple databases (e.g., BFD, MGnify, PDB70, PDB, UniRef30, and UniRef90) to identify related sequences and structural templates. These inputs are fed into the AlphaFold2 neural network, which has been trained using these same databases to learn patterns of protein folding. The model processes the sequence and its evolutionary context to generate a highly accurate predicted protein structure, often rivaling experimentally derived models.

(Illustration generated with assistance from ChatGPT.)
